# Characterization and drug sensitivity profiling of primary malignant mesothelioma cells from pleural effusions

**DOI:** 10.1186/1471-2407-14-709

**Published:** 2014-09-24

**Authors:** Adam Szulkin, Rita Ötvös, Carl-Olof Hillerdal, Aytekin Celep, Eviane Yousef-Fadhel, Henriette Skribek, Anders Hjerpe, László Székely, Katalin Dobra

**Affiliations:** Karolinska Institutet, Department of Laboratory Medicine, Division of Pathology, Karolinska University Hospital F-46, SE-141 86 Stockholm, Sweden; Karolinska Institutet, Department of Microbiology Tumor and Cell Biology (MTC), Nobels väg 16, KI Solna Campus Karolinska Institutet, Box 280, SE 171 77 Stockholm, Sweden

**Keywords:** Malignant mesothelioma, Pleural effusions, Primary cells, Chemotherapy, *Ex vivo* chemosensitivity, Cytotoxic drugs, RRM1, ERCC1 and Individualized treatment

## Abstract

**Background:**

Patients with malignant mesothelioma have a poor prognosis and only 40% respond to first line treatment; a combination of pemetrexed and cisplatin or carboplatin. We used primary malignant mesothelioma cells and an *ex vivo* chemosensitivity assay with future purpose to predict best choice of treatment. The clinical outcome of these patients might be predicted by measuring drug sensitivity.

**Methods:**

Pleural effusions containing primary malignant mesothelioma cells were received from the diagnostic routine. We characterized and tested the chemosensitivity of 18 malignant samples and four benign samples from 16 different patients with pleural effusions. Cells were seeded in a 384-well plate for a robotized *ex vivo* testing of drug sensitivity to 32 different drugs. The primary cells were further characterized by immunocytochemistry to evaluate the proportion of malignant cells and to study the RRM1 and ERCC1 reactivity, two proteins associated with drug resistance.

**Results:**

We observed great individual variability in the drug sensitivity. Primary cell isolates were affected by between one and ten drugs, and resistant to the remaining tested drugs. Actinomycin D and daunorubicin were the two drugs effective in most cases. Adjusting efficiency of individual drugs for varying proportion of tumor cells and to the average effect on benign cells correlated with effect of pemetrexed, cisplatin and survival time. General drug sensitivity, proportion of malignant cells and reactivity to RRM1 correlated to each other and to survival time of the patients.

**Conclusions:**

The proportion of malignant cells and RRM1 reactivity in the pleural effusions correlate to drug sensitivity and survival time. The variability in response to the commonly used chemotherapies emphasizes the need for tests that indicate best individual choice of cytotoxic drugs. The efficiency of the obtained results should preferably be corrected for admixture of benign cells and effects of given drugs on benign cells.

**Electronic supplementary material:**

The online version of this article (doi:10.1186/1471-2407-14-709) contains supplementary material, which is available to authorized users.

## Background

Malignant mesothelioma (MM) is a tumor originating from the mesothelial tissue. The predominant cause is asbestos exposure and therefore the tumor mainly affects the pleura [1, 2]. Accumulation of fluid in the pleural cavity is common in malignant pleural mesothelioma and causes initial symptom as dyspnea [3]. To alleviate symptoms the fluid is drained by pleurocentesis. The collected pleural effusion can be used to establish the diagnosis, based on its content of exfoliated malignant and reactive benign mesothelial cells, inflammatory cells and associated excreted proteins and carbohydrates [4].

MM is a highly therapy resistant tumor with a poor prognosis and the mean overall survival time is 12 months [5]. Chemotherapy is often the only treatment option available but the current first line chemotherapy, a combination of pemetrexed and cisplatin or carboplatin, has a response rate of only 40% and increases patient survival with merely three months [6]. While a number of drug combinations have shown promising results, there is no standardized second line chemotherapy [7]. Thus, in case of treatment failure drugs like doxorubicin and gemcitabine are sometimes used as second line treatment. Patients that respond to chemotherapy have the longest overall survival time, highlighting the importance of accurate drug selection [8].

Excision repair cross-complementing rodent repair deficiency, complementation group 1 (ERCC1) and Ribonucleotide reductase large subunit M1 (RRM1) are two proteins involved in drug resistance. ERCC1 is a main player in the nucleotide excision repair, a DNA repair pathway which has been suggested to clear DNA crosslinks caused by platinum drugs [9]. RRM1 is a subunit of ribonucleotide reductase (RNR), a protein necessary for DNA synthesis. RNR has been shown to be completely inactivated by gemcitabine *in vitro* [10]. Several studies show a link between ERCC1 and RRM1 alone or in combination with other biomarkers to drug sensitivity, progression-free survival or overall survival in lung cancer [11, 12] and in MM [13–15] We have also seen indication that ERCC1 and RRM1 immunoreactivity may explain the sensitivity of MM cell lines to carboplatin [16]. However, these proteins still need further investigation [17].

Hyaluronan and mesothelin are two established biomarkers for MM [18–20]. Levels of these biomarkers have been associated with prognosis, perhaps as an indication of tumor cell differentiation [20, 21]. Hyaluronan is an extracellular matrix polysaccharide involved in cell motility among other processes and is believed to effect tumor aggressiveness [22]. In MM, hyaluronan synthase 1, 2 and 3 are up-regulated and hyaluronan receptors, normally not found on mesothelial cells, are expressed [23, 24]. Mesothelin is a cell membrane protein normally present on mesothelial cells and its exact function is unknown [25–27]. Hyaluronan and mesothelin together can discriminate between metastatic adenocarcinoma and MM with high specificity [18].

The aim of this study was to use primary MM cells in an *ex vivo* chemosensitivity assay with the future purpose to predict the best choice of treatment and predict outcome for individual MM patients. We therefore studied pleural effusions with respect to the drug sensitivity of tumor cells and immunoreactivity of two proteins associated with drug resistance, ERCC1 and RRM1. Simultaneously, effusion supernatants were examined for their content of the diagnostic biomarkers hyaluronan and mesothelin. These results were then correlated to the overall survival time of patients included in this study, assuming that general drug sensitivity associates with a less advanced tumor.

## Methods

### Inclusion criteria and culturing of mesothelioma cells

In this study primary cells from twelve patients diagnosed with malignant mesothelioma, benign mesothelial cells from pleural effusions from four patients with no malignant diagnosis and five MM cell lines were included (for demographic data, see Additional file 1). All effusions but three were received before patient treatment was initiated. All effusions were obtained from the diagnostic routine at the Department of Pathology and Cytology, Karolinska University Hospital in Huddinge, Sweden. The material was collected between 2007 and 2012 and the study was approved by the regional ethics committee in Stockholm.

All MM diagnoses were established by a combination of cytomorphological examination, immunocytochemistry (ICC) and biomarker analysis. The cytomorphological criteria for malignant effusions suggesting mesothelioma are: presence of abnormal cells, high content of cells and cell aggregates, presence of enlarged mesothelial cells, cell engulfment and presence of cells with macronucleoli. The immunocytochemical analysis comprised of staining profile for Epithelial membrane antigen (EMA), Calretinin, HBME-1 and Mesothelin supporting the mesothelial origin of cells, and negative reaction with Carcinoembryonic antigen (CEA), BerEp4 and Thyroid transcription factor-1 excluding a metastatic tumor. High levels of hyaluronan (>75 μg uronic acid/ml) indicates MM [28]. In cases were these analyses were inconclusive Fluorescence in situ hybridization (FISH) was performed, to identify cell population with aneuploidy and/or homozygous deletion of Cyclin-dependent kinase inhibitor 2A (CDKN2A) gene, coding for the p16^INK4A^ protein. In some cases electron microscopy was also used to obtain a correct diagnosis. This approach has been shown to be effective in previous studies by us and others [29–31]. All patients diagnosed with MM were treated with pemetrexed and carboplatin.

All benign effusions were derived from patients with no sign of malignant disease involving the pleural cavity. They included admixture of reactive mesothelial cells and inflammatory cells, without further information of their etiology and without any morphological sign of malignancy. All four patients with benign diagnoses were still alive and without diagnosis of malignancy six months after the collection of fluids.

For culturing of primary cells the effusions were centrifuged at 400 g, 5 min and cells were seeded in Iscove’s modified Dulbecco’s medium (Sigma-Aldrich, St. Louis, USA) containing 20% FBS (Fetal Bovine Serum, Invitrogen, Carlsbad, USA), 0.2% Gentamicin (Invitrogen), 1% Penicillin Streptomycin (Invitrogen) and 1% L-glutamine (Invitrogen). To study the effect of long time culturing on drug sensitivity, two of the samples were cultured up to 18 passages before experiments were performed, with early passages as reference. Two effusions contained substantial amounts of papillary groups; these groups were separated from disassociated cells by shaking the cell flasks after 24 hours, collecting the floating cells and reseeding them in new flasks (summarized in Table 1).

**Table 1:** **Growth properties of primary cell cultures**

Primary cell isolate	Culture identity	Growth property
MMi3	MMi3	Primary cell culture
	MMi4	Grown for eighteen passages
MMi6	MMi6	Primary cell culture
	MMi7	Grown for seven passages
MMi17	MMi17	Adhered disassociated cells
	MMi18	Papillary groups
MMi8	MMi8	Primary cell culture
	MMi9	Seeded the following day
MMi15	MMi15	Papillary groups
	MMi16	Adhered disassociated cells

Primary cells were cultured for several passages, divided into adherent cells and papillary groups or seeded on the following day. MMi6 and MMi17 are two effusions from the same patient.

Five different MM cell lines were included in this study. MM cell lines: STAV-AB, STAV-FCS and ZL-34 cells were kindly provided by Julius Klominek [32, 33]. M-14-K and M-28-K cells were kindly provided by K. Linnainmaa [34]. The STAV-AB cells were grown in Gibco RPMI 1640 medium with 25 mM HEPES buffer (Invitrogen) and 1% L-glutamine and 10% human AB-serum. The STAV-FCS, ZL-34, M-14-K and M-28-K cells were cultured in Gibco RPMI 1640 medium with 25 mM HEPES buffer and 1% L-glutamine, 5% FBS and 5% BS (Bovine Serum, Invitrogen).

### Cytotoxicity assay

The primary mesothelial cells and cell lines were grown to confluency, adherent cells were trypsinized (Invitrogen) and 3000–6000 cells per well were re-seeded in the primary cell culture medium OmniSanguine in a 384-well plate for a robotized *ex vivo* testing of drug sensitivity for 72 hours, as previously described [35, 36]. Briefly, prior to seeding, plates were prepared with 32 different drugs (highest concentrations used summarized in Table 2), distributed to the wells in triplicates and in four different concentrations (diluted 1:1, 1:5, 1:25 and 1:125 in dimethyl sulfoxide) covering a clinically relevant concentration span. Control cells were grown on the same plate, in the same conditions but without the addition of a drug. After 72 hours, VitalDye (Biomarker Ltd, Gödöllő, Hungary) was added to stain living and dead cells, respectively. The amounts of living and dead cells were measured using Qantascope HexascopeHTP automated scanning and analyzing system (Qantascope Biotech, Stockholm, Sweden) [35, 36]. Images were captured using the QantCapture software and amount of living and dead cells was counted by the QantCount software [35–37].

**Table 2:** **Drug concentrations used in cytotoxicity assay**

			Drug concentrations (μg/ml)
Alkylating Agents	Nitrogen mustard	Chlorambucil	83.3
	Aziridine	Mitomycin C	0.3 - 0.6
	Tetrazine	Dacarbazine	16.7
Platinum drugs		Cisplatin	0.2
		Carboplatin	1.7 - 8.3
		Oxaliplatin	4.2
Antimetabolites	Pyrimidine analogues	Fluorouracil	41.7
		Cytarabine	8.3 - 16.7
		Gemcitabine	33.3
	Purine analogues	Mercaptopurine	46.3 - 69.4
		Fludarabine	20.8 - 41.7
		Cladribine	0.8
	Antifolates	Methotrexate	4.2 - 20.8
		Pemetrexed	20.8
	Other	Hydroxyurea	41.7
Antimicrotubule agents	Taxanes	Paclitaxel	3.3
		Docetaxel	8.3 - 16.7
	Vinca alkaloids	Vinblastine	0.2 - 8.3
		Vincristine	0.2 - 0.8
		Vinorelbine	1.7 - 8.3
Topoisomerase inhibitors	Type I	Topotecan	0.2 -1.7
		Irinotecan	16.7
	Type II	Etoposide	16.7
		Amsacrine	4.2
Proteasome inhibitor		Bortezomib	0.2 - 2.9
Multifunctional drugs	Anthracycline	Daunorubicin	4.2 - 16.7
		Doxorubicin	0.8 - 1.7
		Epirubicin	1.7
	Other	Actinomycin D	0.1 - 0.4
		Bleomycin	2.5 - 12.5*
Enzyme		Asparaginase	0.6 - 8.3*
Corticosteroid		Prednisolone	16.7 - 41.7

Drugs are divided according to their mechanism of action. * = IU/ml.

The calculation of the drug efficiency was based on the different degrees of sensitivity at each different concentration, the software calculated by weighted counting of the survival percentage and the drug concentration as follows:


The proportion of tumor cells varied considerably in the different cell isolates. To compensate for this, the above described drug efficiency was corrected according to the proportion of tumor cells present and effect on benign cells (“adjusted drug efficiency”), assuming that the benign cells were affected by the individual drugs in the same magnitude as the corresponding average from the four benign control samples.


Both estimates for drug efficiency were correlated with patient overall survival as surrogate factor, comparing their respective correlation coefficients.

### Immunocytochemistry

Cytospin preparations of primary MM cells were performed on SuperFrost Plus glass slides (Thermo Fisher Scientific Inc, Waltham, MA, USA), fixed in H_2_O with 25% ethanol, 25% methanol, 3% polyethylene glycol (PEG) and stored at −20°C. Before staining, PEG was extracted by decreasing concentrations of ethanol in H_2_O. Immunostaining was performed in a Leica BOND-III automated IHC (see Table 3) with relevant isotype controls, diluted in BOND Primary Antibody Diluent (Leica Microsystems GmbH) and detected with the Bond Polymer Refine Detection kit (Leica Microsystems GmbH) or Bond Polymer Refine Red Detection kit (Leica Microsystems GmbH) according to the manufacturer’s protocol. Briefly, for detection of Desmin, EMA and CD45 slides were pretreated 5 min in a citrate buffer pH 6.0 (Bond Epitope Retrieval Solution 1, Leica Microsystems GmbH), while an EDTA buffer pH 9.0 (Bond Epitope Retrieval Solution 2, Leica Microsystems GmbH) was used for 20 min for ERCC1 and RRM1 staining. Endogenous peroxidase activity was abolished with 3% hydrogen peroxide in H_2_O. Slides were then treated with primary antibodies for 30 min where after secondary IgG was added and incubated for 15 min. Following addition and 15 min incubation with a poly-HRP, bound antibodies were visualized by Diaminobenzidine treatment for 10 min and followed by 10 min counterstain with hematoxylin. Double staining was performed for Desmin and EMA, to distinguish Desmin positive reactive mesothelial cells from malignant cells. EMA was detected as described above whereafter Desmin was detected with a primary antibody, a secondary IgG, incubated with poly-AP and developed with Fast red. All slides were independently evaluated by two experienced cytopathologists (KD and AH) who rated the amount of malignant cells from 0-100% and the staining intensity from 0 to 3 (0 representing no staining and 3 representing strong staining). Discrepant cases were re-evaluated and discussed to reach consensus.

**Table 3:** **Antibodies used in the experiments**

Target	Abbreviation	Antibody	Dilution	Supplier	Product code
Desmin	Desmin	Mouse monoclonal	1:50	1	NCL-DES-DERII
Epithelial membrane antigen	EMA	Mouse monoclonal (clone E29)	1:800	2	M 0613
Leukocyte common antigen	CD45	Mouse monoclonal	1:400	1	NCL-LCA
Excision repair cross-complementing rodent repair deficiency, complementation group 1	ERCC1	Mouse monoclonal Ab-2 (clone 8 F1)	1:200	3	MS-671
Ribonucleotide reductase M1	RRM1	Rabbit polyclonal	1:50	4	Ab81085

Suppliers: 1 = Leica Microsystems GmbH, Wetzlar, 2 = Dako, Glostrup, Denmark, 3 = Thermo Fisher Scientific Inc, Waltham, MA, USA. 4 = Abcam, Cambridge, UK.

### Fluorescence in situ hybridization

In cases where it was difficult to estimate the proportion of malignant cells by immunocytochemistry, we determine this more accurately using the UroVysion bladder cancer kit (Abbott Laboratories, Green Oaks, IL, USA). The kit was used on the primary cell isolates to assess aneuploidy as previously described [30]. Briefly, primary MM cells were incubated in trypsin to dissociate possible cell groups, washed and pellet was spun down on SuperFrost glass slide (Thermo Fisher Scientific Inc). Slides were fixed in acetic acid and methanol, kept 2 min in saline sodium citrate (SSC) solution at 73°C and then incubated 10 min in 0.01 M HCl containing 0.01% Pepsin. After washing the slides the nuclear membranes were stabilized with 1% formaldehyde in 0.01 M MgCl_2_ and dehydrated with increasing concentrations of ethanol. The four directly labeled DNA-probes that hybridize to the centromere region on chromosomes 3, 7 and 17 and to the CDKN2A gene at 9p2, which codes for the p16^INK4A^ protein, were added to the slides, coverslipped, sealed with rubber cement, and placed in a programmed Hybridizer (DAKO) for hybridization. Coverslips and rubber was then removed and slides were stringency washed in SSC with Igepal (Sigma-Aldrich), heated to 73°C, incubated in SSC with Igepal, washed and dried. Finally, cells were stained with 4′,6-diamidino-2-phenylindole and coverslipped. Stainings were evaluated by a cytotechnician (AC) whereby at least 100 cells for each slide were counted, making it possible to determine the proportion of malignant cells. A cell was considered malignant if the number of centromeric probes was increased for at least two of the chromosomes or if fluorescent signal for the CDKN2A gene was missing.

### Biomarkers

Levels of mesothelin (N-ERC) and hyaluronan were measured in the pleural effusions as part of the diagnostic routine and subsets of these results have previously been presented [18]. Measurements were done with Enzyme-Linked Immunosorbent Assays (ELISA) or by High-Performance Liquid Chromatography for hyaluronan, as previously described [18, 28]. For mesothelin the ELISA kit was bought from Immuno-Biological Laboratories Co., Ltd. (Fujioka, Japan, product code 99666/7–16 assay) and for Hyaluronan from Corgenix (Broomfield, CO, USA, product code 029–001).

### Statistical analyses

To examine different possible correlations, linear regression analyses were performed. A Log-rank (Mantel-Cox) test with two-tailed p-values was performed to compare two survival curves in the Kaplan-Meier plot. To further evaluate the effect of drug dilutions in the cytotoxicity assay, sensitivity score was defined as the sum of all drug effects on a primary cell sample, where sensitivity to the highest drug concentration was defined as 1 (1:1), the first dilution as 2 (1:5), the second dilution as 3 (1:25) and the third dilution and lowest concentration as 4 (1:125). Comparisons between subgroups were performed with an unpaired t-test with two-tailed p-values.

## Results

### Great variability in chemosensitivity of primary cell samples

**Figure 1:**
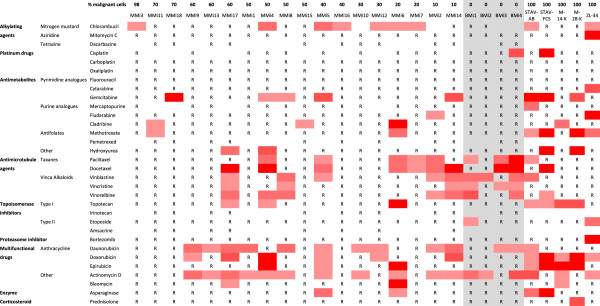
**Chemosensitivity of mesothelioma cells.** Eighteen malignant cell isolates arranged according to their proportion of malignant cells, four benign cell isolates and five cell lines with a pure malignant cell populations to the right. Benign samples in middle on grey background. R = Resistant, more than 50% of cells alive in highest drug concentration. Red color marks a sample where less than 50% of cells were alive. An increasing red color represents sensitivity to an increased drug dilution (1:1, 1:5, 1:25 and 1:125).

The amount of living cells after treatment was normalized to amount of living control cells (Figure 1). All cases with less than 50% dead cells at the highest concentration were defined as resistant. The figure also grades the sensitivity with increasing red color when lower drug concentrations kill more than 50% of the cells. A great individual variability in the drug sensitivity between the different cell cultures was observed. The most resistant cells (MMi3, MMi18, MMi15 and MMi16) were affected by one drug and the most sensitive (MMi17, MMi4, MMi5 and MMi6) by ten drugs. Some drug effects could be seen also on the four benign samples, particularly drugs affecting proliferating cells. The five cell lines displayed a similarly large variability in their drug sensitivity, corresponding with effects seen on primary cells.

Actinomycin D and daunorubicin were the two most effective drugs, affecting 10 of the 18 malignant cell cultures, although some of these drugs also affected two or three of the benign samples. The taxanes (paclitaxel and docetaxel), the vinca alkaloids (vinblastine, vincristine and vinorelbine) and the anthracyclines (daunorubucin, doxorubicin and epirubicin) were the most potent groups of drugs. Surprisingly the effect of bortezomib, cisplatin, carboplatin and pemetrexed was limited, even though pemetrexed was only tested on 50% of the samples.

### The proportion of effective drugs correlated to proportion of malignant cells

**Figure 2:**
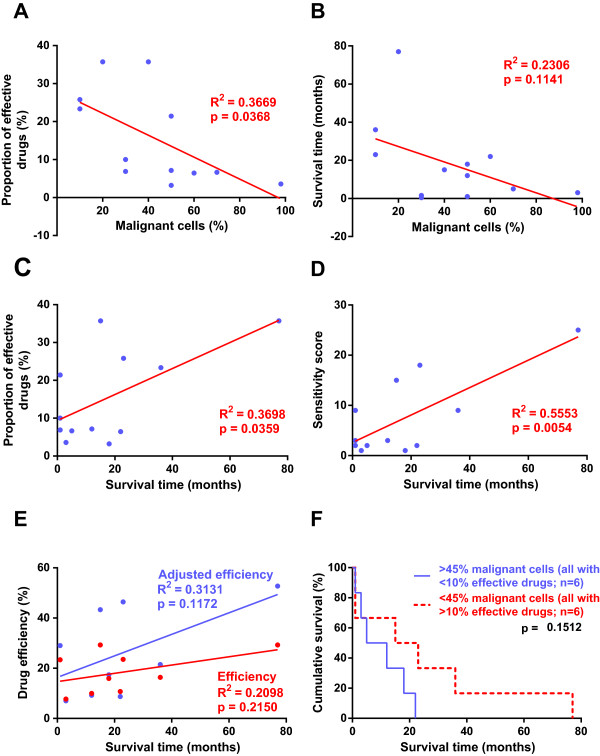
**Correlation between proportion of effective drugs, percent malignant cells and survival time.** Correlations of the proportion of effective drugs, drug efficiency, survival time, percent of malignant cells and sensitivity score plotted in **A**-**D**. Each data point in blue represents one patient, result from the linear regression analysis are presented in red. The drug efficiency is presented in red and adjusted drug efficiency in blue, with data points for each patient and linear regression analysis **(E)**. Kaplan-Meier analysis presents survival time according to proportion of malignant cells in samples and proportion of effective drugs **(F)**. Statistical significance was accepted at p < 0.05 and was seen for proportion of effective drugs and percent of malignant cells **(A)**, proportion of effective drugs and survival time **(C)** and sensitivity score and survival time **(D)**.

The proportion of malignant cells in the primary cell isolates ranged between 10-98% and they inversely correlated to the proportion of effective drugs (p = 0.037) while the correlation to the overall survival of the patients was not statistically significant (Figure 2A-B). The survival time, however, correlated to the proportion of effective drugs (p = 0.036), to the sensitivity score (p = 0.0054) but not to the drug efficiency and adjusted drug efficiency (Figure 2C-E). Kaplan-Meier analysis of proportion of malignant cells and proportion of effective drugs showed a tendency of longer survival time for patients with less malignant cells and with cell isolates effected by more drugs, compared to those with higher amount of malignant cells and lower amount of effective drugs (p = 0.15, Figure 2F).

**Figure 3:**
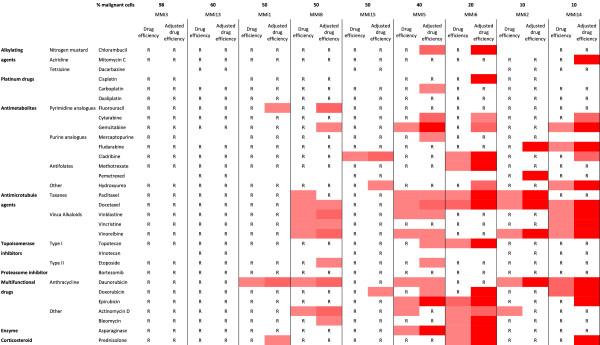
**Drug efficiency and adjusted drug efficiency of primary mesothelioma cells.** Nine malignant cell isolates arranged according to their proportion of malignant cells. R = Resistant, drug efficiency less than 40%. An increasing red color represents increased drug efficiency (40-60%, 60-80% and 80-100%, respectively). Adjusting the drug efficiency to the proportion of malignant cells and drug effect on benign cells; seem to increase the sensitivity of cell isolates and effects of several drugs.

For nine patients, we could study the average efficiency normalized for the varying proportion of malignant cells in the culture and for the effect of the respective drug on the benign control cells. This correction improved the predictive value of drug efficiency for survival time (p = 0.12, Figure 2E). Similar adjustment for each drug increased the explanatory values for efficiency, using survival time as correlating surrogate factor. The sensitivity profile changed accordingly (Table 4 and Figure 3). Carboplatin, cisplatin and pemetrexed affected only one malignant cell sample each, while gemcitabine and doxorubicin affected four isolates each. Adjusting the drug efficiency for pemetrexed, cisplatin and doxorubicin increased the tendency to correlate with survival, with increased coefficient of determination and decreased p-values (p = 0.09, p = 0.05 and p = 0.17, respectively).

**Table 4:** **Correlations of drug efficiency and adjusted drug efficiency with survival**

	Drug efficiency vs. survival		Adjusted drug efficiency vs. survival	
	R ^2^	p-value	R ^2^	p-value
Pemetrexed	0.36	0.40	0.84	0.09
Chlorambucil	0.14	0.37	0.42	0.08
Hydroxyurea	0.08	0.47	0.32	0.11
Cisplatin	0.46	0.14	0.67	0.05
Doxorubicin	0.05	0.55	0.25	0.17
Paclitaxel	0.24	0.18	0.40	0.07
Asparaginase	0.17	0.27	0.32	0.11
Etoposide	0.00	0.95	0.14	0.32
Docetaxel	0.28	0.14	0.39	0.07
Vinblastine	0.00	0.98	0.08	0.46
Cytarabine	0.08	0.47	0.15	0.30
Vincristine	0.01	0.79	0.07	0.49
Gemcitabine	0.00	0.99	0.05	0.57
Bleomycin	0.37	0.08	0.41	0.06
Cladribine	0.37	0.08	0.38	0.08
Fludarabine	0.01	0.85	0.01	0.81
Oxaliplatin	0.04	0.59	0.04	0.59
Dacarbazine	0.33	0.42	0.33	0.42
Fluorouracil	0.04	0.58	0.04	0.60
Carboplatin	0.05	0.56	0.03	0.63
Topotecan	0.56	0.02	0.54	0.03
Bortezomib	0.05	0.55	0.03	0.64
Vinorelbine	0.03	0.67	0.00	0.96
Methotrexate	0.60	0.01	0.57	0.02
Actinomycin D	0.20	0.23	0.14	0.32
Daunorubicin	0.13	0.34	0.07	0.48
Mercaptopurine	0.11	0.47	0.05	0.63
Prednisolone	0.48	0.04	0.42	0.06
Epirubicin	0.36	0.09	0.22	0.20
Mitomycin C	0.29	0.13	0.05	0.57

For each individual drug a linear regression analysis was performed comparing survival time and drug efficiency or adjusted drug efficiency. The explanatory value (R^2^) and p-values are sorted according to the largest gain in explanatory value when adjusting the drug efficiency. Statistically significant departure of the slope from 0 was accepted at p < 0.05 and were found for prednisolone and drug efficiency as well as for topotecan and methotrexate, both for drug efficiency and when adjusting the drug efficiency.

### Cytoplasmatic staining of RRM1 correlated to proportion of effective drugs

**Figure 4:**
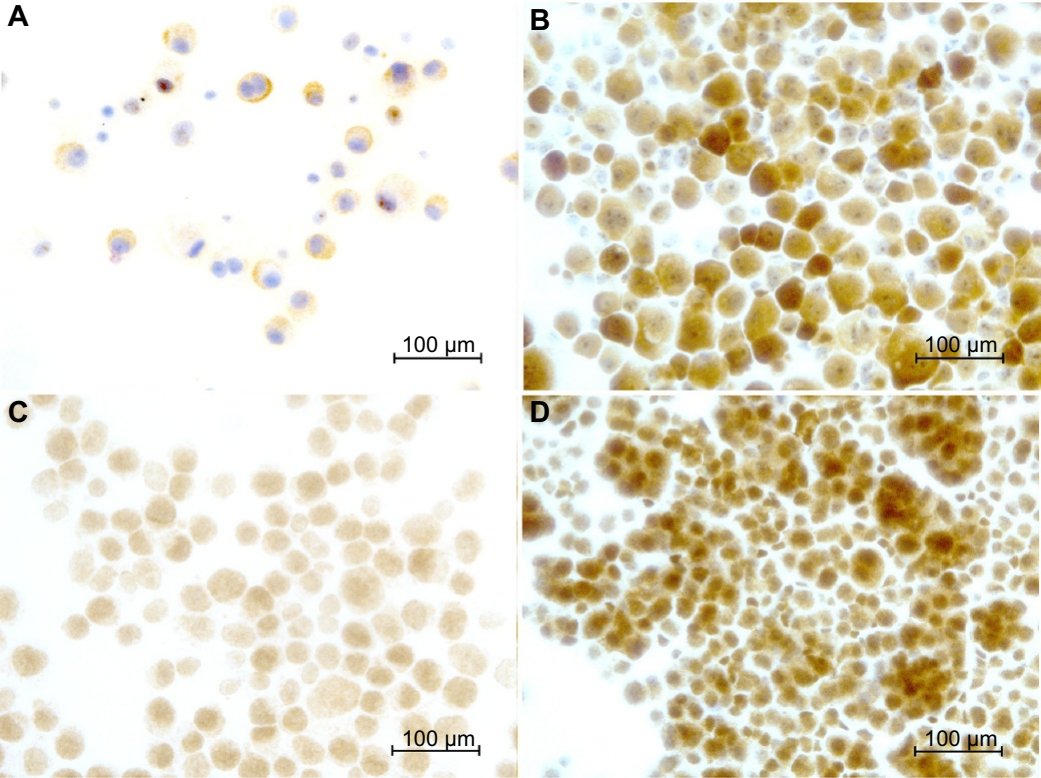
**Immunoreactivity of RRM1 and ERCC1 in primary malignant mesothelioma cells.** The panel shows representative micrographs: **(A)** weak RRM1 staining intensity (score 1) **(B)** strong RRM1 staining intensity (score 3) **(C)** weak ERCC1 staining intensity (score 1) and **(D)** strong ERCC1 staining intensity (score 3), respectively. Scale bar = 100 μm.

**Figure 5:**
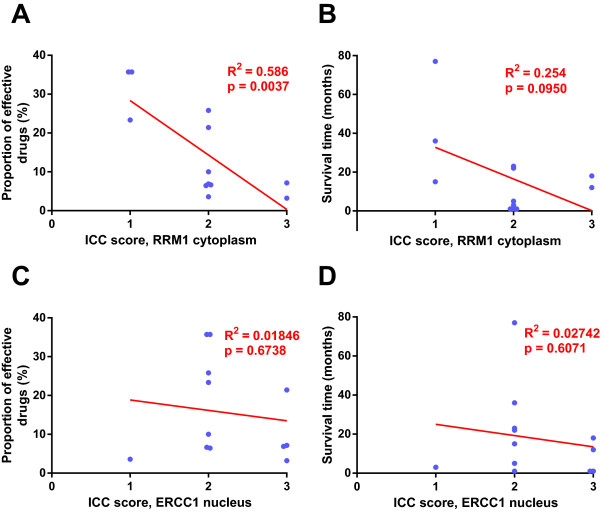
**RRM1 and ERCC1 immunoreactivity correlated to proportion of effective drugs and survival time. (A)** Proportion of effective drugs plotted against RRM1 staining. **(B)** Survival time plotted against RRM1. **(C)** Proportion of effective drugs plotted against ERCC1 staining. **(D)** Survival time plotted against ERCC1 Regression analysis presented in red, data points for each patient in blue. Statistically significant departure of the slope from 0 was accepted at p < 0.05 and seen for proportion of effective drugs and RRM1 immunoreactivity **(A)**.

All samples were stained for RRM1 and ERCC1 (Figure 4A-D), evaluating the staining of the malignant cell populations. RRM1 reactivity strongly correlated to the number of effective drugs (p = 0.0037) but not to the survival time (Figure 5A-B). Primary cell cultures with a higher ICC score for RRM1 were more resistant to the antimicrotubule agents (except paclitaxel), topotecan, hydroxyurea, gemcitabine, methotrexate, bleomycin and doxorubicin, compared to those with a lower ICC score. ERCC1 staining was not correlated to the proportion of effective drugs or the survival time of the patients (Figure 5C-D).

### Hyaluronan and mesothelin

Hyaluronan and mesothelin values varied greatly between the different patients and different samples. Hyaluronan levels correlated to the RRM1 cytoplasmatic staining of the twelve patient samples (p = 0.024) but not to the survival time (Additional file 2A-B). No correlation was found for mesothelin (Additional file 2C).

### Aneuploidy and p16^INK4A^ deletion

FISH was performed on nine cases and cells with aneuploidy were found in all of them. In six of these a homozygous deletion of 9p21 band could be demonstrated, but presence of this did not correlate to patient data, drug sensitivity of cells or RRM1 and ERCC1 staining.

### Proportion of malignant cells correlates to RRM1 staining and *ex vivo*drug effect

When dividing the results into two groups, according to the amount of malignant cells, the cytoplasmatic staining of RRM1 was stronger in the group with the higher amount of malignant cells (p = 0.022). The sensitivity score and proportion of effective drugs was higher in the group with the lower proportion of malignant cells (p = 0.0425 and p = 0.027). A split of the results into two groups, according to proportion of effective drugs, presented a significant difference between the two groups, were the proportion of malignant cells was lower and the RRM1 cytoplasmatic staining weaker in the group with the sensitive samples (p = 0.017 and p = 0.022). Dividing the results in two groups, according to levels of hyaluronan, resulted in a significant difference in the RRM1 cytoplasmatic reactivity, stronger in the group with the higher hyaluronan values (p = 0.022). With a similar categorization according to mesothelin levels, no correlation was found.

When separating the results into two groups, according to the survival time or according to the sensitivity score, no significant correlations were found. The six samples with more malignant cells showed no correlation to patient data, drug effect, RRM1 or ERCC1 staining.

### Comparison between cell isolates from the same patient

The importance of how cultures were isolated and possible change during tumor progression was studied (Table 1). When a primary culture (MMi3) was kept for 18 passages (culture MMi4) we could see a decrease in the RRM1 staining, while ERCC1 staining remained. Simultaneously the proportions of effective drugs increased from 4% to 36%.

Similarly MMi6, obtained from a patient with long term survival, was also tested after seven passages as MMi7. A second sample was obtained from the same patient four years later. This second cell isolate was separated in two fractions, the first (MMi17) consisting of cells adhering after overnight culture and the second (MMi18) representing poorly adhering papillary groups. Similarly another sample was grown out as adhering or papillary cell groups (MMi16 and MMi15, respectively).

The early isolates (MMi6 and MMi7) showed the same RRM1 and ERCC1 staining; also here the drug sensitivity was lost after prolonged culture. When comparing MMi17 to MMi18 the proportion of malignant cells and RRM1 staining was similar, while the ERCC1 reactivity was higher and the number of effective drugs much lower in the isolate consisting of papillary tumor fragments (only one compared to ten for the primarily adhering cells). Corresponding differences were not seen, comparing the MMi15 and MMi16 cell isolates.

When looking at all four isolates from this patient, the proportion of malignant cells increased from 20% to 60-70% four years later. The RRM1 staining also increased, while ERCC1 of adherent cells decreased. The amount of effective drugs was high in MMi6 and MMi17 and low in MMi7 and MMi18. The biomarker levels (hyaluronan and mesothelin) were largely increased during the four years between the samples.

To test the effect of delayed seeding of cells, an aliquot of MMi8 was also seeded the following day (MMi9, Table 1). When comparing these two cultures we could see a decrease in number of effective drugs (from six to three), while the reactivity to RRM1 and ERCC1 remained unchanged.

MMi15 and MMi16 are from the same primary cell culture where MMi15 was prepared from papillary groups and MMi16 from adhered disassociated cells (Table 1). These two preparations contained similar amounts of tumor cells, had similar reactivity to RRM1 and ERCC1, and similar resistance to the tested drugs.

## Discussion

Chemotherapeutical treatment of malignant mesothelioma continues to be struggling and even though several different agents and drug combinations have been suggested, the combination of pemetrexed and cisplatin or carboplatin remains the first line treatment [6, 38–40]. The 40% response rate of these treatments is, however, disappointing and patients responding to treatment have the longest survival time [8]. This demonstrates the need for greater understanding of patient response, treatment effects and markers correlating to these aspects. In cell lines we have previously seen large differences in sensitivity to different drugs, and in immunoreactivity of different predictive markers, suggesting a possibility for personalized treatment [16, 41]. By performing more detailed studies of MM patient’s primary malignant cells we might be able to individualize treatment to increase response rates and survival times.

In this study we perform an extensive characterization of primary cells from pleural effusions from patients with malignant mesothelioma. We observe a large individual variability in their drug sensitivity and a widespread resistance (Figures 1, 2A and 3), reflecting the clinical situation with limited effect of chemotherapy and a highly individual response rate. The patient survival time correlated to the proportion of effective drugs and sensitivity scores (Figure 2C-F) and seems to be influenced by the proportion of malignant cells and RRM1 reactivity (Figures 2B,F and 5B). RRM1 staining correlated to general drug sensitivity of primary cells (Figure 5A), comparable findings have been seen in non-small cell lung cancer patients treated with cisplatin and vinorelbine, where immunohistochemical evaluation of RRM1 predicted drug effect [42]. The observed variability in chemosensitivity seems to partly depend on the proportion of malignant cells in the samples and RRM1 levels but other factors associated with individual tumor heterogeneity are probably also important.

Among drugs used in first and second line treatment, cisplatin, carboplatin and pemetrexed showed little or no effect on any of the tested malignant cell isolates, whereas gemcitabine and doxorubicin, affected five and six of the malignant isolates, respectively. The limited effect of pemetrexed might be explained by our previous findings, that cell cycle distribution is a more sensitive approach when detecting short term effect of this drug [16]. After correcting for admixture of benign cells and the average effect of respective drug on the benign cell cultures, the correlation of drug efficiency to survival improved. Interestingly, this correlation was most prominent with pemetrexed and cisplatin, the combination which is today’s first line treatment for MM. However, several phase II studies have achieved comparable results using pemetrexed and carboplatin [43, 44] as well as the combination of carboplatin, liposomized doxorubicin and gemcitabine [45]. These five drugs affect different cell samples (Figure 3) which indicates that some of the patients not responding to pemetrexed and cisplatin treatment might have had a better response if they were treated with another drug combination. Although not yet proven, this kind of *ex vivo* testing of drug sensitivity may provide a basis for personalized choice of treatment.

Long-term culturing of primary cells may affect the response to drug exposure. In one sample, culturing over 18 passages changed the RRM1 reactivity and increased the general sensitivity to drugs (Figures 2A and 5A), perhaps associated to sub-cloning. Such an effect was not seen in a second case grown for seven passages. When separating cells into disassociated cells and papillary groups, the latter were less sensitive in one sample (Table 1 and Figure 1). Culturing patient samples for several passages or dividing them into disassociated cells and papillary groups thus may affect the outcome of testing, indicating the importance to use primary cultures from entire samples.

Pleural effusions obtained from the same patient at four years interval (Table 1 and Figure 1) show an increased proportion of malignant cells in the sample. The cells had increased RRM1 reactivity over time and drug resistance (Figures 2A-C and 5A), perhaps reflecting a progressed disease.

The proportion of malignant cells in the effusions seems to have a decisive effect in these experiments, correlating to proportion of effective drugs and affecting the survival time (Figure 2A, B and F). When we adjust the efficiency for the proportion of malignant cells and possible effects on benign cells, we can still see that patient samples with a lower proportion of malignant cells are more sensitive to the different drugs (Figures 1 and 3). These results indicate that pleural effusions from patients with MM where the proportions of malignant cells are higher might reflect a more advanced disease, with shorter survival time and extensive drug resistance. The observed cytotoxic effects on cell lines corresponded to the effects seen on primary cells, verifying that these cell lines are a suitable model system for studying primary MM cells.

In a few of the primary cell cultures the used drug concentrations varied (cf. Table 2). This did, however, not affect the sensitivity of the primary cells, except when using fludarabine and topotecan, which showed increased effects at the higher concentration. ERCC1 staining was not shown to correlate to any of the measured factors. One cause for this may be that the included drugs do not affect the DNA directly, since ERCC1 mainly indicates resistance to DNA affecting drugs.

## Conclusions

Our experimental approach allows a simultaneous determination of *ex vivo* chemosensitivity of primary malignant mesothelioma cells to 32 different drugs. The results demonstrate that there are large differences in drug sensitivity between the different primary cell isolates. Interestingly, drug sensitivity, amount of malignant cells and RRM1 reactivity seemed to correlate to each other and to the survival time of the patients. To evaluate the efficiency of individual drugs results should be adjusted for the proportion of malignant cells, i.e., for the possible effect on benign cells in the culture. After this correction, correlations were seen for pemetrexed and cisplatin effects and survival time. We could also see that these two drugs affect different cell samples compared to carboplatin, doxorubicin and gemcitabine, accentuating the use of these three drugs when first line treatment fails.

The proportion of malignant cells in the pleural effusions from patients with MM also seems to play an important role in drug sensitivity and survival time. These results indicate that higher amount of malignant cells in pleural effusions are seen in patients with an advanced disease, shorter survival time and extensive drug resistance.

The variability in response to the commonly used therapeutic alternatives emphasizes the need for tests that would indicate best individual choice of drugs. Drug sensitivity assays of cells isolated from an effusion like the one tested here may provide such an opportunity. Confounding factors that must be dealt with concerns the admixture of benign cells and how these cells react to drug exposure. The trends seen here will be further evaluated in larger patient cohorts and the reactivity pattern will be correlated to the effect of the clinically administered drugs.

## Electronic supplementary material

Additional file 1: **Demographic data: Age- and gender distribution of effusions subjected for cytotoxic drugs.** (PDF 35 KB)

Additional file 2: **Correlations with hyaluronan and mesothelin.** Survival time and RRM1 staining plotted against levels of hyaluronan and mesothelin. Each data point in blue represents a patient, presented together with results from the linear regression analyses in red. Statistical significance was accepted at p < 0.05 and was seen for RRM1 staining and levels of hyaluronan. (PDF 48 KB)

## Abbreviations

MM: Malignant mesothelioma
ERCC1: Excision repair cross-complementing rodent repair deficiency, complementation group 1
RRM1: Ribonucleotide reductase large subunit M1
RNR: Ribonucleotide reductase
ICC: Immunocytochemistry
EMA: Epithelial membrane antigen
CEA: Carcinoembryonic antigen
FISH: Fluorescence in situ hybridization
CDKN2A: Cyclin-dependent kinase inhibitor 2A
FBS: Fetal Bovine Serum
BS: Bovine Serum
PEG: Polyethylene glycol
ELISA: Enzyme-Linked Immunosorbent Assays.
