# Supplementary material for: Characterization and drug sensitivity profiling of primary malignant mesothelioma cells from pleural effusions
Source: BMC Cancer. 2014 Sep 24;14:709. doi: 10.1186/1471-2407-14-709 (PMC4190467; doi:10.1186/1471-2407-14-709)
Supplement: Supplementary file 1 — Additional file 1: Demographic data: Age- and gender distribution of effusions subjected for cytotoxic drugs. (PDF 35 KB) [file 12885_2014_4896_MOESM1_ESM.pdf]

Additional file 1

Pleural effusions	Number of patients	Male (%)	Female (%)	Age, median (IQR)
Benign	4	0 (0)	4 (100)	59 (45-76)
Malignant mesothelioma	12	12 (100)	0 (0)	69 (58-87)
