# Supplementary figures and images for: Characterization and drug sensitivity profiling of primary malignant mesothelioma cells from pleural effusions
Source: BMC Cancer. 2014 Sep 24;14:709. doi: 10.1186/1471-2407-14-709 (PMC4190467; doi:10.1186/1471-2407-14-709)

Additional file 2, Szulkin et al

A.

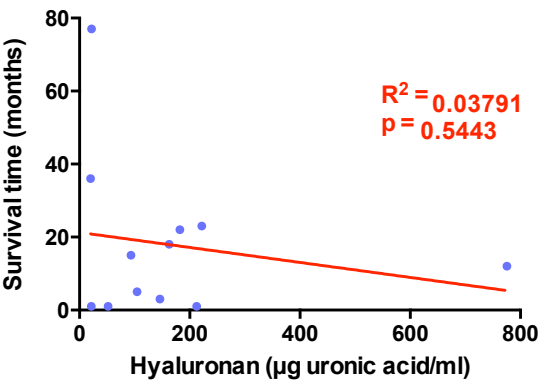

B.

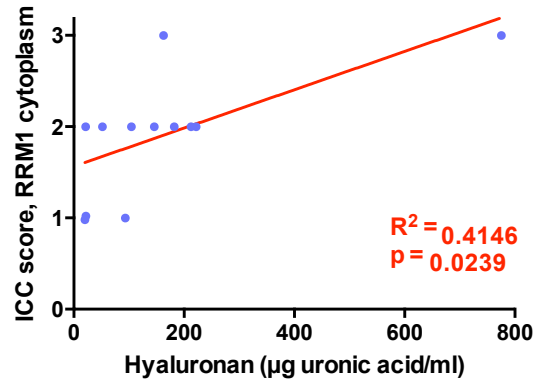

C.

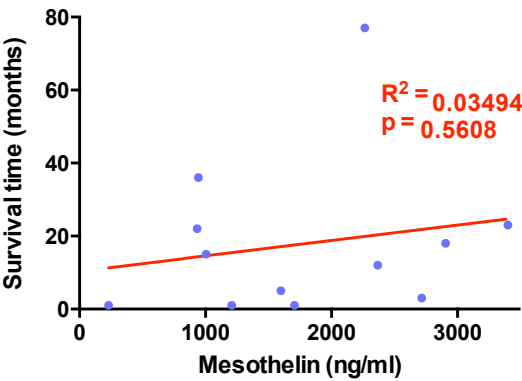

Supplement: Supplementary file 2 — Additional file 2: Correlations with hyaluronan and mesothelin. Survival time and RRM1 staining plotted against levels of hyaluronan and mesothelin. Each data point in blue represents a patient, presented together with results from the linear regression analyses in red. Statistical significance was accepted at p < 0.05 and was seen for RRM1 staining and levels of hyaluronan. (PDF 48 KB) [file 12885_2014_4896_MOESM2_ESM.pdf]
